# Post-Mortem Examination—Rupture of the Intestine without External Laceration

**Published:** 1852-10

**Authors:** N. S. Davis

**Affiliations:** Chicago


ART. IV.—*Post Mortem Examination—Rupture of the Intestine without external laceration.* By N. S. DAVIS, M. D.

ON the 14th September, 1852, I was directed by the Coroner to make a *post mortem* examination, in connection with Prof. A. B. Palmer, of the body of Mr. F., aged abont 50 years. The history of the case is briefly as follows :

Mr. F. was the keeper of a saloon, or drinking shop, on South Water-street, and between two and three days before his death he had an altercation with one of his drinking customers, in which he was thrown down and received one or more severe *kicks* from his antagonist directly against the abdomen. Symptoms of most intense peritoneal inflammation immediately followed, accompanied by vomiting, extreme prostration, and death in little more than 48 hours after the injury. On examining the body, six hours after death, we found no external wound, either lacerated or penetrating, nor was there any ecchymosis or discoloration of the skin ; but the abdominal parietes appeared swollen and tense.

A longitudinal incision was made from the ensiform cartilage to the pubes, through the linea alba, and another from the crest of one ilium to that of the other. There was little or no appearance of infiltration into the cellular tissue covering the abdominal muscles, but on raising the flaps made by the incisions, the peritoneal membrane lining the abdominal muscles was found inflamed throughout its whole extent, but much more intensely across the inguinal and pubic regions than above. The folds of the same membrane constituting the omentum were also strongly injected with blood, and in a few points adherent to the abdominal walls, by means of recently effused coagulable lymph. The peritoneal covering of the small intestines was very generally inflamed, and in the left inguinal region it was extensively covered with coagulable lymph, intensely red, and adherent to the surface lining the abdominal muscles. On breaking these adhesions, all of which were very recent, a fold of the small intestine lying about one inch above the internal inguinal ring, was found ruptured and its contents discharged into the peritoneal cavity. The rupture or orifice in the intestine was about three-quarters of an inch in length. The peritoneal surfaces in the immediate vicinity of this opening were intensely inflamed and glued together by recently effused lymph. A considerable portion of the intestine was inverted, and the mucous membrane examined; which was found to present, at some points, appearances of moderate inflammation of recent origin, but no marks of ulceration or other more chronic disease. The other abdominal viscera appeared healthy.

I have been induced to put this case on record, because, so far as I recollect, a direct rupture of the intestines by blows on the abdomen, inflicted by a blunt instrument like a man's foot, without leaving any well marked appearances of violence on the external surface, is of sufficiently rare occurrence to be interesting in a medico-legal point of view.

CHICAGO, *September* 24, 1852.

